# Defining, conceptualizing, and measuring perceived maternal care quality in low- to high-income countries: a scoping review protocol

**DOI:** 10.1186/s13643-021-01608-6

**Published:** 2021-02-24

**Authors:** Kyrah K. Brown, Godfred O. Boateng, Peace Ossom-Williamson, Laura Haygood

**Affiliations:** 1 Department of Kinesiology, University of Texas at Arlington, 500 W. Nedderman Drive, Box 19407, Arlington, TX 76019-0407 USA; 2 Research Data Services, UTA Libraries, University of Texas at Arlington, 702 Planetarium Place, Arlington, TX 76019 USA; 3 Gibson D. Lewis Health Science Library, University of North Texas Health Science Center, 3500 Camp Bowie Blvd, Fort Worth, TX 76107 USA

**Keywords:** Scoping review, Maternal mortality, Quality of care, Measurement

## Abstract

**Background:**

Health practitioners and researchers must be able to measure and assess maternal care quality in facilities to monitor, intervene, and reduce global maternal mortality rates. On the global scale, there is a general lack of consensus on how maternal care quality is defined, conceptualized, and measured. Much of the literature addressing this problem has focused primarily on defining, conceptualizing, and measuring clinical indicators of maternal care quality. Less attention has been given in this regard to perceived maternal care quality among women which is known to influence care utilization and adherence. Therefore, there is a need to map the literature focused on defining, conceptualizing, and measuring perceived maternal care quality across low-, middle-, and high-income country contexts.

**Methods:**

This scoping review protocol will follow the Arksey and O’Malley methodological framework. A comprehensive search strategy will be used to search for articles published from inception to 2020 in Ovid MEDLINE, Embase, AMED, and WHO Global Index Medicus. Gray literature will be included. Two independent reviewers will screen articles by title and abstract, then by full-text based on pre-determined inclusion/exclusion criteria. A third reviewer will arbitrate any discrepancies. This protocol outlines a four-step analytic approach that includes numerical, graphical, tabular, and narrative summaries to provide a comprehensive description of the body of literature.

**Discussion:**

The findings from this scoping review will provide a comprehensive overview of the existing evidence on perceived maternal care quality. The findings are expected to inform future work on building consensus around the definition and conceptualization of perceived maternal care quality, and lay the groundwork for future research aimed at developing measures of perceived maternal care quality that can be applied across country contexts. Consequently, this review may aid in facilitating coordinated efforts to measure and improve maternal care quality across diverse country contexts (i.e., low-, middle-, and high-income country contexts).

**Review registration:**

This scoping review has been registered in the Open Science Framework (osf.io/k8nqh).

**Supplementary Information:**

The online version contains supplementary material available at 10.1186/s13643-021-01608-6.

## Background

In the last 25 years, the global maternal mortality ratio (MMR) declined nearly 44% [1]. Despite this global decline, the burden of maternal mortality differs considerably by country economic development status and geographic region [1]. For example, low- and middle-income countries (LMICs) account for approximately 99% of global maternal deaths [1]. Among high-income countries (HICs), MMR rates steadily declined from 1990 to 2015, with the USA having the highest rates of maternal deaths compared to its peer HICs [2]. In 2015, all United Nations member countries adopted the 2030 Agenda for Sustainable Development [3]. The agenda outlines 17 Sustainable Development Goals (SDGs) and 169 targets that represent a call to action for all countries—developed and developing—to “ensure healthy lives and promote well-being for all at all ages” [3]. In particular, the aim of SDG Target 3.1 is to “reduce the global maternal mortality ratio to less than 70 maternal deaths per 100,000 live births by 2030” [3]. Available evidence suggests that if all participating countries meet SDG Target 3.1, the lives of an estimated 1.6 million women could be saved [4]. There is growing consensus that targeting maternal care quality (MCQ) will be a necessary approach to achieving the SDG Target 3.1 [5–7]. While there has been extensive work focused on developing and examining clinical performance indicators of MCQ (e.g., cesarean delivery for low-risk nulliparious women; receipt of postpartum care within 8 weeks after delivery), less is known about patient-reported measures of MCQ, which can be useful for monitoring and improving factors known to influence women’s health outcomes and maternal care utilization behaviors [8]. Also, a focus on perceived MCQ is crucial because women’s experiences in the maternal care continuum (prenatal, intrapartum, and postpartum care) can influence care seeking and utilization of essential, potentially life-saving maternal care services [5, 9–12].

In order to achieve SDG Target 3.1 and reduce the global burden of maternal mortality, health practitioners and researchers must be able to continuously measure and assess perceived MCQ. There is, however, no standard definition, conceptualization, or measurement of perceived MCQ. An example of a contemporary MCQ definition is “the degree to which maternal health services for individuals and populations increase the likelihood of timely and appropriate treatment for the purpose of achieving desired outcomes that are both consistent with current professional knowledge and uphold basic reproductive rights” [13]. Moreover, several frameworks have been developed or adapted to conceptualize MCQ [13–17]. An example is Donabedian’s model for examining and evaluating three components of care quality: structure (e.g., material resources, human resources, organizational structure), process (e.g., what is done in giving and receiving care), and outcomes (e.g., effects of care on the health status of patients and populations) [15]. Furthermore, several challenges exist regarding the measurement of perceived MCQ. For example, many studies documenting existing measures of MCQ have been largely focused on the monitoring of provider- or facility-reported indicators, with little emphasis on patients’ experience of care [8, 18, 19]. In fact, little is known about existing validated instruments designed to measure perceived MCQ among women. Also, perceived MCQ has been measured at distinct stages of the maternal care continuum (prenatal, intrapartum, and postpartum care) which may provide a disconnected understanding of women’s perceptions regarding the quality of care received.

Overall, there appears to be great heterogeneity in the way in which perceived MCQ is defined, conceptualized, and measured. This heterogeneity may limit generalizability across country contexts and maternal care contexts [14, 20]. Prior empirical reviews have focused primarily on provider- or facility-reported measures of MCQ [8, 18] or primarily low- or middle-income country contexts only [21–23]. A comprehensive knowledgebase regarding MCQ may be needed to facilitate coordinated efforts to measure and improve the provision of maternal care across diverse country economic contexts (i.e., low-, middle-, and high-income contexts). In turn, countries may be able to make progress toward achieving SDG Target 3.1, and subsequently reduce the global burden of maternal mortality.

Thus, the objective of this review is to systematically map the literature on the definition, conceptualization, and measurement of perceived MCQ across low-, middle-, and high-income countries. To ensure a comprehensive understanding of perceived MCQ, this review will focus on the maternal care continuum including prenatal care, intrapartum care, and postpartum care. Scoping review is the chosen approach because its objective is to provide a comprehensive overview of the available literature rather than critically appraise individual studies [24]. Also, given the heterogeneous and “globally siloed” nature of the literature on perceived MCQ, a scoping review is needed to map and summarize the literature in a systematic manner. In doing so, the findings from this scoping review can inform the development of more focused research questions. More specifically, the findings may inform future work focused on building consensus around the definition and conceptualization of MCQ and lay the groundwork for future research aimed at developing comprehensive and context-specific measures of perceived MCQ that are cross culturally relevant. Consequently, this work has the potential to guide efforts to improve MCQ, and ultimately make progress toward achieving SDG Target 3.1.

## Method

### Design and registration

The review protocol has been registered within the Open Science Framework database [25] and is being reported in accordance with the guidance provided in the Preferred Reporting Items for Systematic Reviews and Meta-Analyses extension for Scoping Reviews (PRISMA-ScR) and the PRISMA extension for protocols (PRISMA-P) [26] (see PRISMA-P checklist in Supplemental File 1). The Arksey and O’Malley five-stage framework for conducting scoping reviews was used for this review [27]. This framework stipulates the following steps: (1) identifying the research question, (2) identifying relevant studies, (3) study selection, (4) charting the data, and (5) collating, summarizing, and reporting the results.

#### Stage 1: Identifying the research question

This scoping review is guided by two research questions:

RQ^1^: How has perceived maternal care quality been defined in low-, middle-, and high-income countries?

RQ^2^: How has perceived maternal care quality been conceptualized and measured in low-, middle-, and high-income countries?

#### Stage 2: Identifying relevant studies (eligibility criteria)

Eligible studies will be selected according to the Population-Concept-Context (PCC) framework recommended by the Joanna Briggs Institute as shown in Table 1 [24]. This review will be guided by the World Health Organization’s (WHO) definition of women of childbearing age (15-49). Also, while the timeframe of antenatal care and intrapartum care tends to be consistent across countries, the recommended period of postpartum (or postnatal) care may vary across countries. Therefore, this review will include studies focused on postpartum care to women up to 1 year after delivery—which falls within the WHO’s surveillance period for maternal mortality. Low-, middle-, and high-income countries will be determined by the World Bank list of economies, which has classified the countries according to their economic status [28, 29]. This review will have no time period restrictions. The authors recognize that the English language is not the universal language of science. However, due to the study team’s lack of financial and language resources (e.g., funding, professional translators), only articles published in the English language or with an English-language translation available will be included. Prior research suggests that reviews that are compliant with standard reporting guidelines and use English-language restrictions do not contribute to systematic bias or demonstrate low credibility [30, 31].

**Table 1:** PCC framework

Criteria	Description
**Population**	• Women (and girls) of childbearing age (15-49) • Pregnant women and girls (up to 40 weeks gestation regardless of pregnancy outcome) • Postpartum women and girls (up to 1 year post-delivery)
**Concept**	• All study designs focused on measuring the concept of “perceived maternal care quality”—women’s perceptions regarding the safety, effectiveness, timeliness, equity, and patient-centeredness of their antenatal, intrapartum, or postpartum care received in a medical facility
**Context**	• Articles published in all countries regardless of income classification • Evidence published up until recently • Articles published in English language or with an English-language translation available

This review will include articles from the inception of each respective database to 2020. This review will include experimental studies, quasi-experimental studies, analytical observational studies (including prospective and retrospective cohort, case-control, and cross-sectional studies), qualitative and quantitative studies focused on the measurement of perceived MCQ, systematic and scoping reviews, and meta-analysis papers. To avoid publication bias, unpublished studies and gray literature will be included in this review. This review will exclude editorials, letters, and case reports because these sources will not provide empirical evidence needed to answer the present review’s research questions.

##### Inclusion criteria

To be included, articles must meet the following criteria:

- Article includes women and girls of childbearing age (15-49)
- Evidence published up to recent
- Studies conducted in low-, middle-, or high-income country
- All articles will be included irrespective of their study designs
- Gray literature resources include government and non-governmental organization reports and academic dissertations

##### Exclusion criteria

Articles reporting the following will be excluded:

- Articles that do not include women and girls of childbearing age (15-49)
- Articles that do not measure women (and girls) perceptions of maternal care quality
- Articles that only measure clinical/objective indicators of maternal care quality
- Articles focused on measuring maternal care quality outside of a health facility (e.g., home births)

#### Stage 3: Study selection (search strategy)

The research team will collaboratively identify the key terms for the search strategies, and the research librarians will develop initial strategies utilizing input from all authors and test for maximum sensitivity, keeping in mind specificity. Specificity will be calculated by dividing the total number of records identified by the number of relevant records identified. The research librarians will build the search strategy focusing on four major concepts: pregnancy and its outcomes and complications, maternal health care, quality of care, patient satisfaction, and assessment tools. For each concept, the research librarians will truncate keywords as necessary and include relevant subject headings to achieve a comprehensive set of citations. For the purposes of this protocol, the research team used the aforementioned steps to develop a draft central search strategy and had the draft central search strategy reviewed by an expert medical librarian at a different institution to provide feedback to help refine the strategy. The draft central search strategy is provided in Table 2.

**Table 2:** . Draft Central Search Strategy

Ovid MEDLINE Search	
1. Postpartum Period/ or Pregnancy/ or Labor, Obstetric/ or Pregnancy, High-Risk/ or Labor, Obstetric/ or Fetus/ or Breast Feeding/ or Parturition/ or Birth Setting/ or Natural Childbirth/ or Term Birth/ or Pregnancy Outcome/ or Abortion, Spontaneous/ or Live Birth/ or Stillbirth/ or Pregnancy, Multiple/ or Pregnancy, Quadruplet/ or Pregnancy, Quintuplet/ or Pregnancy, Triplet/ or Pregnancy, Twin/ or Pregnancy, Unplanned/ or Pregnancy Complications/ or Abortion, Spontaneous/ or Abortion, Threatened/ or Chorea Gravidarum/ or Diabetes, Gestational/ or Fetal Death/ or Fetal Diseases/ or Hypertension, Pregnancy-Induced/ or Morning Sickness/ or Nuchal Cord/ or Obesity, Maternal/ or Obstetric Labor Complications/ or Oligohydramnios/ or Pelvic Floor Disorders/ or Pemphigoid Gestationis/ or Perinatal Death/ or Phenylketonuria, Maternal/ or Placenta Diseases/ or Polyhydramnios/ or Pregnancy Complications, Cardiovascular/ or Pregnancy Complications, Hematologic/ or Pregnancy Complications, Infectious/ or Pregnancy Complications, Neoplastic/ or Pregnancy In Diabetics/ or Pregnancy, Ectopic/ or Pregnancy, Prolonged/ or Prenatal Injuries/ or Puerperal Disorders/ or Pregnancy Trimesters/ or Pregnancy Trimester, First/ or Pregnancy Trimester, Second/ or Pregnancy Trimester, Third/ or Pregnant Women/ 2. (parturition or obstetric* or prenatal or postnatal or perinatal or maternal or pregnan* or childbirth or birth or birthing or births or antenatal or gestation* or antepartum or postpartum or labor or labour or preterm or pre-term or stillbirth or neonatal or neo-natal or intra-partum or intrapartum or fetal or foetal or fetus* or foetus* or induction* or induce or trimester).ti,kw. 3. 1 or 2 4. “Health Care Quality, Access, and Evaluation”/ or After-Hours Care/ or Answering Services/ or Culturally Competent Care/ or Delivery of Health Care, Integrated/ or Health Care Reform/ or Healthcare Disparities/ or Telemedicine/ or Safety-net Providers/ or Hospitalization/ or “Referral and Consultation”/ or Office Visits/ or House Calls/ 5. (healthcare or care or consultation or consultations or office or appointment* or hospital or hospitals or clinic or clinics or monitor* or center or centers or centre or centres or unit or units or facilit* or exam or exams or provider or providers or practitioner or practitioner or induction or induce or referral or surgery or surgeries or surgical or procedure).ti,kw. 6. 4 or 5 7. Perinatal Care/ or Postnatal Care/ or Maternal Health Services/ or Maternal-Child Health Services/ or Prenatal Care/ or exp Obstetric Surgical Procedures/ 8. 7 not Abortion, Induced/ 9. (3 and 6) or 8 10. Quality of Health Care/ or Health Services Accessibility/ or Patient Satisfaction/ or Patient Preference/ or Personal Satisfaction/ 11. ((patient* or prenatal or postnatal or perinatal or maternal or maternity or pregnan* or mother* or parent* or wom?n* or childbirth or birth or births or antenatal or breastfe* or eclampsia or preeclampsia or pre-eclampsia or gestation* or antepartum or postpartum or labor or labour or preterm or pre-term or stillbirth or neonatal or neo-natal or intra-partum or intrapartum) adj3 (quality or satisf* or experience* or perception* or perceived or perspective* or opinion* or memory or memories or fear or trauma*)).ti,kw. 12. 10 or 11 13. Evaluation Studies as Topic/ or “Surveys and Questionnaires”/ or Health Care Surveys/ or Patient Reported Outcome Measures/ or Patient Health Questionnaire/ or Self Report/ or exp "Outcome and Process Assessment, Health Care"/ or Factor Analysis, Statistical/ or Psychometrics/ 14. (assessment or feedback* or questionnaire* or interview* or tool* or instrument* or measur* or survey or surveys or focus group* or scale* or validity or reliability or EFA or “factor analysis”).ti,kw. 15. 13 or 14 16. (exp animals/ not humans.sh.) or Veterinarians/ or exp Veterinary Medicine/ 17. (9 and 12 and 15) not 16 18. limit 17 to english language	

The research librarians will modify the strategies so that they are appropriate for each database. The review team plans to search for peer-reviewed publications in the following databases from inception to 2020: Ovid MEDLINE, Embase, AMED, WHO Global Index Medicus, Grey: BioMed Central Journals, Google Scholar, and ProQuest Dissertations and Theses. The World Health Organization International Clinical Trials Registry Platform (ICTRP) will not be used because it is currently closed indefinitely to non-WHO staff due to high use during COVID-19 pandemic.

The research librarians will collect and upload metadata from all identified records to EndNote and remove duplicates. This review will use a two-stage screening process as a large number of studies are expected. The first screening phase will consist of reviewing the titles and abstracts of each record and removing articles that are irrelevant. The second screening phase will consist of reviewing the full text of included articles. After reading each paper, further irrelevant articles will be removed from the study sample. The remaining articles will have relevance to the study question and will be put forward for data extraction. Each stage of this process will be completed by two reviewers (KB and GB) independently with disagreement resolved initially by consensus and if needed by a third reviewer. A summary of the study selection process will be presented as a PRISMA flowchart. A table of excluded studies and reasons for exclusion will be provided.

#### Stage 4: Charting the data (data extraction)

To ensure the most relevant and comprehensive information is extracted in a systematic way, we will create a data extraction table in Microsoft Word. As shown in Table 3, the extraction of charting elements will be based on guidance from the JBI Review’s Manual [32] and additional columns relevant to this review’s research questions using a format illustrated in other protocols [33, 34]. We will record additional result details including (1) database searched, (2) search date, (3) search string with limiters, (4) results retrieved, and (5) number of duplications removed. The data extraction tool will be piloted by two reviewers on two articles first and where needed, modifications will be made to the data extraction tool. However, there may be further refinements added to include any relevant data that was not initially included during the extraction process. Data from all included studies will then be charted by the first reviewer (KB) and extraction checked by the second reviewer (GB).

**Table 3:** Preliminary table of charting elements and relevant questions for data

Chart elements	Associated questions
**Publication details**	
Author(s)	Who wrote the article?
Year of publication	What year was the article published?
Country of origin	Where was the study/document conducted and/or published?
Country economic classification	Is the country of origin classified as low-, middle-, or high-income?
Publication type	What type of publication is this? (empirical study, gray literature)
**Study/article details**	
Aims/purpose	What were the aims of the study/article?
Methodological design	What methodological design was used?
Type of care	Did this study/article focus on antenatal, intrapartum, or postpartum care?
Structure and process of care	Does the study/article describe the structure and process of care as it relates to the country context?
Study population and sample size	Who was the target population and how many were included in the study/article?
Methods	What specific methods were used?
Construct(s) measured	What constructs were measured? What were the study measures?
Intervention	Was an intervention used in this study? (yes or no)
Comparator and duration of the intervention (if applicable)	If yes, what was the comparator and duration of the intervention?
Outcomes (if applicable)	If yes, what was the intervention outcome?
**Conceptualization and measurement details**	
What is perceived maternal care quality?	How is perceived maternal care quality defined or conceptualized?
Theoretical/Conceptual Framework	What theory or framework was used to conceptualize maternal care quality in this study/article?
Donabedian concept category	Which of the following Donabedian concept categories are relevant to the constructs measured in this study/article? (process, structure, outcomes)
How is perceived maternal care measured?	What type of instrument was used for measurement? (e.g., questionnaire, scaling, interview guide) If applicable, what level of measurement was used (nominal, ordinal, interval, ratio) to measure perceived maternal care quality?
Number of instrument items	How many items were included in the instrument?
Instrument dimensions	What dimensions are measured or conceptualized?
Number of dimensions	How many dimensions are included?
Instrument psychometric properties	Was the instrument validated? What was the reliability statistic? What was the content validity?
Limitations and challenges	Were there any reported limitations or quality issues (not a critical appraisal)?
Recommendations for further study	Were there any recommendations for further study?

#### Stage 5: Collating, summarizing, and reporting results

This stage will consist of four major steps that include the use of numerical, graphical, tabular, and narrative summaries. In the first analytic step, the review will provide a descriptive numerical and tabular summary that includes the total number of included articles, including a breakdown of articles by year of publication, country economic classification (low, middle, and high income), type of care (antenatal, intrapartum, and postpartum), type of study design, and type of instrument used (qualitative or quantitative).

The Arksey and O’Malley methodology recommends adopting a framework to collate and summarize the extracted data in a systematic manner [27]. Therefore, the review results will utilize country economic classification (low, middle, high income) and Donabedian’s model of care quality (structure, process, outcome) to organize the results in a structured, systematic manner, and thereby enhance the articulation of the results [27, 32]. The second analytic step will aim to answer the first research question (*RQ*^*1*^*: How has perceived maternal care quality been defined in low-middle-, and high-income countries?*). This review will present a table (see Table 4 in Supplemental File 2), organized by country economic classification, to report the following information for each included article: author/year, country name, sample description, type of care (antenatal, intrapartum, postpartum), definition of perceived maternal care quality, theoretical framework used to conceptualize maternal care quality from the patient’s perspective. Based on this table, a written narrative will describe similarities and differences in the way in which perceived maternal care quality has been defined within each country economic classification and across the three different classifications. This written narrative will also describe identified gaps in the literature.

The third analytic step will aim to answer the second research question (*RQ*^*2*^*: How has perceived maternal care quality been conceptualized and measured in low-, middle-, and high-income countries?*). First, this review will include a chart (see Figure 1 in Supplemental File 2) to visually map the number of articles by country economic classification (low, middle, high income) and by Donabedian concept category (process, structure, outcome). If a single article focuses on constructs that fall within two or more Donabedian concept categories, each one will be counted separately. The purpose of this chart is to illustrate trends and patterns in the overarching maternal care quality concepts measured overall and by country economic classification.

Second, this review will present a table (see Table 5 in Supplemental File 2), organized by Donabedian’s concept categories. This table will report the following information for each article: author/year, country income classification, construct(s) measured, type of care (antenatal, intrapartum, and postpartum), and type of instrument used. This table is intended to provide a more detailed breakdown of the information presented in Figure 1. The intent of this table is not to assess the quality of the article or instruments used. Based on this table, a narrative summary will be organized according to the three Donabedian concept categories. For each Donabedian concept category, the written narrative will (a) summarize patterns in the types of constructs measured in the overall literature and by country income classification and (b) patterns in the types of constructs measured by type of care and by country income classification. The narrative summary will also describe any identified gaps.

## Discussion

This scoping review will provide a comprehensive view of the existing evidence on perceived MCQ. This review’s proposed methodology for identifying and mapping the existing literature includes a rigorous and transparent approach, documented in such a way to promote replication [27]. The objective of this scoping review is to map the literature on the definition, conceptualization, and measurement of perceived MCQ across low-, middle-, and high-income countries. As the first known scoping review of its kind, the findings from this scoping review are expected to inform future work focused on building consensus around the definition and conceptualization of perceived MCQ and lay the groundwork for future research aimed at developing measures of perceived MCQ that can be applied across diverse country economic contexts. This scoping review has the potential to lay the foundation for future efforts to make progress toward achieving SDG Target 3.1, and subsequently reducing the global burden of maternal mortality.

## Supplementary Information

**Additional file 1:.** PRISMA-P (Preferred Reporting Items for Systematic review and Meta-Analysis Protocols) 2015 checklist: recommended items to address in a systematic review protocol* .

**Additional file 2:.** Table 4. Summary of articles that provide a definition of perceived MCQ by country economic classification. Figure 1. Summary of articles by Donabedian concept measured and country economic classification (sample data). Table 5. Summary of articles’ conceptualization and measurement of perceived MCQ by Donabedian concept category and country economic classification.

## Data Availability

Not applicable.

## Abbreviations

AXIS: Appraisal tool for cross-sectional studies
HIC: High-income country
JBI: Joanna Briggs Institute
LMIC: Low- and middle-income country
MCQ: Maternal care quality
MMR: Maternal mortality ratio
PRISMA-P: Preferred Reporting Items for Systematic Review and Meta-Analysis Protocols
SDG: Sustainable Development Goals

## References

[1] World Health Organization, UNICEF, United Nations, Department of Economic and Social Affairs, Population Division, World Bank. Trends in maternal mortality: 1990 to 2015: estimates by WHO, UNICEF, UNFPA, World Bank Group and the United Nations Population Division [Internet]. 2015 [cited 2018 Nov 8]. Available from: http://www.who.int/reproductivehealth/publications/monitoring/maternal-mortality-2015/en/

[2] Kassebaum NJ, Barber RM, Bhutta ZA, Dandona L, Gething PW, Hay SI (2016). Global, regional, and national levels of maternal mortality, 1990–2015: a systematic analysis for the Global Burden of Disease Study 2015. The Lancet..

[3] Transforming our world: the 2030 Agenda for Sustainable Development .:. Sustainable Development Knowledge Platform [Internet]. [cited 2020 Jul 2]. Available from: https://sustainabledevelopment.un.org/post2015/transformingourworld

[4] McArthur JW, Rasmussen K, Yamey G. How many lives are at stake? Assessing 2030 sustainable development goal trajectories for maternal and child health. BMJ [Internet]. British Medical Journal Publishing Group; 2018 [cited 2020 Jun 27];360. Available from: https://www.bmj.com/content/360/bmj.k373 10.1136/bmj.k373 PMC5813301 29449222

[5] Sudhinaraset M, Afulani P, Diamond-Smith N, Bhattacharyya S, Donnay F, Montagu D. Advancing a conceptual model to improve maternal health quality: the person-centered care framework for reproductive health equity. Gates Open Res [Internet]. 2017 [cited 2020 Jun 27];1. Available from: https://www.ncbi.nlm.nih.gov/pmc/articles/PMC5764229/10.12688/gatesopenres.12756.1 PMC5764229 29355215

[6] Akachi Y, Tarp F, Kelley E, Addison T, Kruk ME (2016). Measuring quality-of-care in the context of sustainable development goal 3: a call for papers. Bull World Health Organ..

[7] Austin A, Langer A, Salam RA, Lassi ZS, Das JK, Bhutta ZA (2014). Approaches to improve the quality of maternal and newborn health care: an overview of the evidence. Reprod Health..

[8] Saturno-Hernández PJ, Martínez-Nicolás I, Moreno-Zegbe E, Fernández-Elorriaga M, Poblano-Verástegui O (2019). Indicators for monitoring maternal and neonatal quality care: a systematic review. BMC Pregnancy Childbirth..

[9] Vedam S, Stoll K, Taiwo TK, Rubashkin N, Cheyney M, Strauss N (2019). The Giving Voice to Mothers study: inequity and mistreatment during pregnancy and childbirth in the United States. Reprod Health..

[10] McLemore MR, Altman MR, Cooper N, Williams S, Rand L, Franck L (2018). Health care experiences of pregnant, birthing and postnatal women of color at risk for preterm birth. Soc Sci Med..

[11] Sharma J, Leslie HH, Kundu F, Kruk ME (2017). Poor quality for poor women? Inequities in the quality of antenatal and delivery care in Kenya. PLOS ONE. Public Library of Science.

[12] Davis D-A (2019). Obstetric racism: the racial politics of pregnancy, labor, and birthing. Med Anthropol. Routledge.

[13] Hulton LA, Mathews Z, Stones RW (2000). A framework for the evaluation of quality of care in maternity services.

[14] Benova L, Moller A-B, Hill K, Vaz LME, Morgan A, Hanson C (2020). What is meant by validity in maternal and newborn health measurement? A conceptual framework for understanding indicator validation. PLOS ONE Public Library of Science.

[15] Donabedian A (1988). The quality of care: how can it be assessed?. JAMA. American Medical Association.

[16] Lane DS, Kelman HR (1975). Assessment of maternal health care quality: conceptual and methodologic issues. Med Care..

[17] World Health Organization. WHO recommendations on intrapartum care for a positive childbirth experience. World Health Organization; 2018. 30070803

[18] Brizuela V, Leslie HH, Sharma J, Langer A, Tunçalp Ö (2019). Measuring quality of care for all women and newborns: how do we know if we are doing it right? A review of facility assessment tools. Lancet Glob Health..

[19] Moller A-B, Newby H, Hanson C, Morgan A, El Arifeen S, Chou D, et al. Measures matter: a scoping review of maternal and newborn indicators. PLoS ONE [Internet]. 2018 [cited 2020 Dec 31];13. Available from: https://www.ncbi.nlm.nih.gov/pmc/articles/PMC6177145/10.1371/journal.pone.0204763 PMC6177145 30300361

[20] Moucheraud C, McBride K. Variability in health care quality measurement among studies using service provision assessment data from low- and middle-income countries: a systematic review. The American Journal of Tropical Medicine and Hygiene; 2020;tpmd190644. 10.4269/ajtmh.19-0644 PMC7470535 32588806

[21] Tripathi V (2016). A literature review of quantitative indicators to measure the quality of labor and delivery care. Int J Gynecol Obstet..

[22] Pirkle CM, Dumont A, Zunzunegui M-V (2011). Criterion-based clinical audit to assess quality of obstetrical care in low- and middle-income countries: a systematic review. Int J Qual Health Care. Oxford Academic.

[23] Tripathi V, Stanton C, Strobino D, Bartlett L (2019). Measuring the quality of maternal and care processes at the time of delivery in sub-Saharan Africa: development and validation of a short index. BMC Pregnancy Childbirth. BioMed Central.

[24] Peters MDJ, Marnie C, Tricco AC, Pollock D, Munn Z, Alexander L (2020). Updated methodological guidance for the conduct of scoping reviews. JBI Evid Synth..

[25] Brown KK, Boateng GO, Williamson PO, Haygood L. Patient-reported measures for the assessment of maternal care quality: a scoping review protocol. OSF; 2020 [cited 2020 Jul 8]; Available from: https://osf.io/k8nqh

[26] Moher D, Shamseer L, Clarke M, Ghersi D, Liberati A, Petticrew M (2015). Preferred reporting items for systematic review and meta-analysis protocols (PRISMA-P) 2015 statement. Syst Rev..

[27] Arksey H, O’Malley L (2005). Scoping studies: towards a methodological framework. Int J Soc Res Methodol. Routledge.

[28] Fantom N, Serajuddin U. The World Bank’s classification of countries by income [Internet]. World Bank, Washington, DC; 2016 [cited 2020 Dec 23]. Available from: http://hdl.handle.net/10986/23628

[29] The World Bank. Countries and economies [Internet]. 2020 [cited 2020 Dec 23]. Available from: https://data.worldbank.org/country

[30] Morrison A, Polisena J, Husereau D, Moulton K, Clark M, Fiander M (2012). The effect of English-language restriction on systematic review-based meta-analyses: a systematic review of empirical studies. Int J Technol Assess Health Care..

[31] Wang Z, Brito JP, Tsapas A, Griebeler ML, Alahdab F, Murad MH (2015). Systematic reviews with language restrictions and no author contact have lower overall credibility: a methodology study. Clin Epidemiol..

[32] Peters M, Godfrey C, Khalil H, Mcinerney P, Soares C, Parker D. 2017 Guidance for the conduct of JBI scoping reviews. 2017.

[33] Nittas V, Mütsch M, Ehrler F, Puhan MA (2018). Electronic patient-generated health data to facilitate prevention and health promotion: a scoping review protocol. BMJ Open..

[34] Gilfoyle M, MacFarlane A, Salsberg J (2020). Conceptualising, operationalising and measuring trust in participatory health research networks: a scoping review protocol. BMJ Open..

