# Supplementary material for: Defining, conceptualizing, and measuring perceived maternal care quality in low- to high-income countries: a scoping review protocol
Source: Syst Rev. 2021 Feb 24;10:61. doi: 10.1186/s13643-021-01608-6 (PMC7903867; doi:10.1186/s13643-021-01608-6)
Supplement: Supplementary file 1 — Additional file 1:. PRISMA-P (Preferred Reporting Items for Systematic review and Meta-Analysis Protocols) 2015 checklist: recommended items to address in a systematic review protocol* . [file 13643_2021_1608_MOESM1_ESM.doc]

**PRISMA-P (Preferred Reporting Items for Systematic review and Meta-Analysis Protocols) 2015 checklist: recommended items to address in a systematic review protocol***

| Section and topic | Item No | Checklist item | Description |
| --- | --- | --- | --- |
| ADMINISTRATIVE INFORMATION | | |  |
| Title: |  |  | The authors identify the document as a scoping review protocol in the title. |
| Identification | 1a | Identify the report as a protocol of a systematic review |  |
| Update | 1b | If the protocol is for an update of a previous systematic review, identify as such |  |
| Registration | 2 | If registered, provide the name of the registry (such as PROSPERO) and registration number | The authors provide the name of the registry and number/link in the abstract and in the manuscript (under design and registration. |
| Authors: |  |  | The authors have listed this information in the manuscript on the title page and at the end of the manuscript in accordance with journal guidelines. |
| Contact | 3a | Provide name, institutional affiliation, e-mail address of all protocol authors; provide physical mailing address of corresponding author | In accordance with journal submission guidelines, author contributions is listed at the end of the manuscript. |
| Contributions | 3b | Describe contributions of protocol authors and identify the guarantor of the review |  |
| Amendments | 4 | If the protocol represents an amendment of a previously completed or published protocol, identify as such and list changes; otherwise, state plan for documenting important protocol amendments | The authors included a statement about their plans for documenting protocol amendments at the end of the manuscript under “Protocol Amendments” |
| Support: |  |  |  |
| Sources | 5a | Indicate sources of financial or other support for the review | In accordance with the submission/format guidelines, information about financial support and other disclosures is presented at the end of the manuscript. |
| Sponsor | 5b | Provide name for the review funder and/or sponsor |  |
| Role of sponsor or funder | 5c | Describe roles of funder(s), sponsor(s), and/or institution(s), if any, in developing the protocol |  |
| INTRODUCTION | | |  |
| Rationale | 6 | Describe the rationale for the review in the context of what is already known | The authors described the rationale for the review in the context of what is already known in the Background section (Lines 80-87; 107-115 |
| Objectives | 7 | Provide an explicit statement of the question(s) the review will address with reference to participants, interventions, comparators, and outcomes (PICO) | The authors provide an explicit statement of the review objective (Lines 116-118). Also using the Arksey and O’Malley methodological framework, the authors present the research questions (Lines 140-145) and use the Population-Concept-Context framework most appropriate for scoping reviews (Lines 170-171) |
| METHODS | | |  |
| Eligibility criteria | 8 | Specify the study characteristics (such as PICO, study design, setting, time frame) and report characteristics (such as years considered, language, publication status) to be used as criteria for eligibility for the review | The authors document the eligibility criteria (Lines 146-186) |
| Information sources | 9 | Describe all intended information sources (such as electronic databases, contact with study authors, trial registers or other grey literature sources) with planned dates of coverage | The authors describe the information sources in great detail with planned dates of coverage (Lines 162-169; and 201-2017) |
| Search strategy | 10 | Present draft of search strategy to be used for at least one electronic database, including planned limits, such that it could be repeated | The authors present the search strategy (Lines 189-218) and in Table 2 |
| Study records: |  |  |  |
| Data management | 11a | Describe the mechanism(s) that will be used to manage records and data throughout the review | The authors document the record management (Line 209); the section process (Lines 209-215), data collection process (Lines 226-237), and data items (Table 3). |
| Selection process | 11b | State the process that will be used for selecting studies (such as two independent reviewers) through each phase of the review (that is, screening, eligibility and inclusion in meta-analysis) |  |
| Data collection process | 11c | Describe planned method of extracting data from reports (such as piloting forms, done independently, in duplicate), any processes for obtaining and confirming data from investigators |  |
| Data items | 12 | List and define all variables for which data will be sought (such as PICO items, funding sources), any pre-planned data assumptions and simplifications |  |
| Outcomes and prioritization | 13 | List and define all outcomes for which data will be sought, including prioritization of main and additional outcomes, with rationale | This is not applicable to scoping review |
| Risk of bias in individual studies | 14 | Describe anticipated methods for assessing risk of bias of individual studies, including whether this will be done at the outcome or study level, or both; state how this information will be used in data synthesis | This is not applicable to scoping review. |
| Data synthesis | 15a | Describe criteria under which study data will be quantitatively synthesised | In accordance with the Arksey and O’Malley methodology, the authors descript the steps that will be used to collate, summarize and report the results (Lines 240-281). |
|  | 15b | If data are appropriate for quantitative synthesis, describe planned summary measures, methods of handling data and methods of combining data from studies, including any planned exploration of consistency (such as I^2^, Kendall’s τ) |  |
|  | 15c | Describe any proposed additional analyses (such as sensitivity or subgroup analyses, meta-regression) |  |
|  | 15d | If quantitative synthesis is not appropriate, describe the type of summary planned |  |
| Meta-bias(es) | 16 | Specify any planned assessment of meta-bias(es) (such as publication bias across studies, selective reporting within studies) | This is not applicable to scoping review |
| Confidence in cumulative evidence | 17 | Describe how the strength of the body of evidence will be assessed (such as GRADE) | This is not applicable to scoping review |

*** It is strongly recommended that this checklist be read in conjunction with the PRISMA-P Explanation and Elaboration (cite when available) for important clarification on the items. Amendments to a review protocol should be tracked and dated. The copyright for PRISMA-P (including checklist) is held by the PRISMA-P Group and is distributed under a Creative Commons Attribution Licence 4.0.**

*From: Shamseer L, Moher D, Clarke M, Ghersi D, Liberati A, Petticrew M, Shekelle P, Stewart L, PRISMA-P Group. Preferred reporting items for systematic review and meta-analysis protocols (PRISMA-P) 2015: elaboration and explanation. BMJ. 2015 Jan 2;349(jan02 1):g7647.*
