# Supplementary material for: Defining, conceptualizing, and measuring perceived maternal care quality in low- to high-income countries: a scoping review protocol
Source: Syst Rev. 2021 Feb 24;10:61. doi: 10.1186/s13643-021-01608-6 (PMC7903867; doi:10.1186/s13643-021-01608-6)
Supplement: Supplementary file 2 — Additional file 2:. Table 4. Summary of articles that provide a definition of perceived MCQ by country economic classification. Figure 1. Summary of articles by Donabedian concept measured and country economic classification (sample data). Table 5. Summary of articles’ conceptualization and measurement of perceived MCQ by Donabedian concept category and country economic classification. [file 13643_2021_1608_MOESM2_ESM.docx]

**Table 4. Summary of articles that provide a definition of perceived MCQ by country economic classification**

| ***Low-Income Countries*** | | | | | |
| --- | --- | --- | --- | --- | --- |
| **Author/Year** | **Country Name** | **Sample Description** | **Type of Care** | **MCQ Definition** | **Theoretical Framework** |
|  |  |  |  |  |  |
| ***Middle-Income Countries*** | | | | | |
| **Author/Year** | **Country Name** | **Sample Description** | **Type of Care** | **MCQ Definition** | **Theoretical Framework** |
|  |  |  |  |  |  |
| ***High-Income Countries*** | | | | | |
| **Author/Year** | **Country Name** | **Sample Description** | **Type of Care** | **MCQ Definition** | **Theoretical Framework** |
|  |  |  |  |  |  |

**Figure 1. Summary of articles by Donabedian concept measured and country economic classification (sample data)**

**Table 5. Summary of articles’ conceptualization and measurement of perceived MCQ by Donabedian concept category and country economic classification**

| ***Donabedian Concept: Process (i.e., article measures factors related to the quality of care provision processes)*** | | | | | |
| --- | --- | --- | --- | --- | --- |
| **Author/Year** | **Country Income Classification** | **Type of Care** | **Construct(s)**  **Measured** | **Instrument Type (qual. or quant.)** | **Reliability and Content Validity (if applicable)** |
|  |  |  |  |  |  |
| ***Donabedian Concept: Structure (i.e., article measures factors related to the quality of material and human resources and organizational structure characteristics)*** | | | | | |
| **Author/Year** | **Country Income Classification** | **Type of Care** | **Construct(s) Measured** | **Instrument Type (qual. or quant.)** | **Reliability and Content Validity (if applicable)** |
|  |  |  |  |  |  |
| ***Donabedian Concept: Outcome (i.e., article measures the effects of care on the health status of patients)*** | | | | | |
| **Author/Year** | **Country Income Classification** | **Type of Care** | **Construct(s) Measured** | **Instrument Type (qual. or quant.)** | **Reliability and Content Validity (if applicable)** |
|  |  |  |  |  |  |
